# Orbital Apex Syndrome Secondary to Herpes Zoster Ophthalmicus: A Case of Irreversible Optic Nerve Damage

**DOI:** 10.7759/cureus.46522

**Published:** 2023-10-05

**Authors:** Martha Plasencia, Brent R McQueen

**Affiliations:** 1 Medical Education, Berkeley Eye Center, Kingwood, USA; 2 Ophthalmology, Berkeley Eye Center, Kingwood, USA

**Keywords:** ophthalmoplegia, optic neuropathy, varicella-zoster virus, orbital apex syndrome, herpes zoster ophthalmicus

## Abstract

Herpes zoster ophthalmicus (HZO) arises because of the reactivation of latent varicella-zoster virus (VZV) infection in the trigeminal nerve ganglion and typically presents with ocular manifestations. However, infrequent neurological complications can also be seen. Rarely occurring, orbital apex syndrome presents itself as a severe complication of HZO, characterized by the involvement of multiple cranial nerves and vascular structures within the orbital apex. A 70-year-old male patient being followed for HZO developed orbital apex syndrome after 14 days of treatment, resulting in ophthalmoplegia, vision loss, and significant morbidity. Clinical diagnosis was confirmed by magnetic resonance imaging of the orbit. The treatment involved a combination of systemic steroid and antiviral therapy, resulting in a favorable resolution of ophthalmoplegia within six months. However, optic-neuropathy-induced vision loss was irreparable. Early recognition, antiviral therapy, and multidisciplinary management are crucial in preventing permanent visual impairment and neurological deficits associated with this uncommon complication.

## Introduction

Herpes zoster ophthalmicus (HZO) is characterized as the herpes zoster (HZ) infection affecting the ophthalmic branch of the trigeminal nerve, ranking as the second most prevalent form of HZ, following thoracic zoster [1,2]. In 20% to 70% of patients with HZO, ocular symptoms can be seen, leading to potential complications such as lid scarring, conjunctivitis, neurotrophic keratitis, episcleritis, ophthalmoplegia, acute retinal necrosis, glaucoma, and optic deterioration [2,3]. Neurological complications are less common in comparison to ocular complications and may include postherpetic neuralgia, ophthalmoplegia, optic neuritis, ptosis, and rarely orbital apex syndrome (OAS) [3,4]. Of the various infectious etiologies, HZO is a rare cause of OAS [4].

The most common clinical features of a disease process in the orbital apex are vision loss and painful, limited eye movements. Typically, ocular manifestations begin one to four weeks after the appearance of skin lesions [5]. OAS has been characterized as a condition involving impairment to the third cranial nerve (oculomotor nerve), the fourth cranial nerve (trochlear nerve), the sixth cranial nerve (abducens nerve), and the ophthalmic branch of the fifth cranial nerve (V1), along with dysfunction of the second cranial nerve (optic nerve) [6]. This case report highlights the importance of early diagnosis and prompt intervention to optimize outcomes in patients with OAS secondary to HZO.

## Case presentation

A 70-year-old male presented to our ophthalmology clinic after being treated for an HZ infection for two weeks. On examination, the patient presented with blistering skin lesions and crusted vesicles with redness in the right forehead, periorbital, and brow region. He complained of pain with irritation and was unable to open his right eye, which presented with ophthalmoplegia evidenced by 1+ ptosis, immobility of the eye and pupil, and loss of accommodation. The best visual acuity was finger counting in the right eye (OD) and 20/25 in the left eye (OS). Direct light reflex showed a positive afferent pupillary defect (APD) in the right eye and anisocoria with a fixed and dilated pupil. Intraocular pressure (IOP) measurement and dilation of the right eye were deferred. On slit-lamp examination, the right eye exhibited superficial punctate keratitis, low-grade iritis, and conjunctivitis. Additionally, edema and 1+ cells in the anterior chamber were observed. On fundoscopic examination, the cup-to-disc ratio was 0.3, the optic nerve was pink, and the macula appeared normal. 

**Figure 1:**
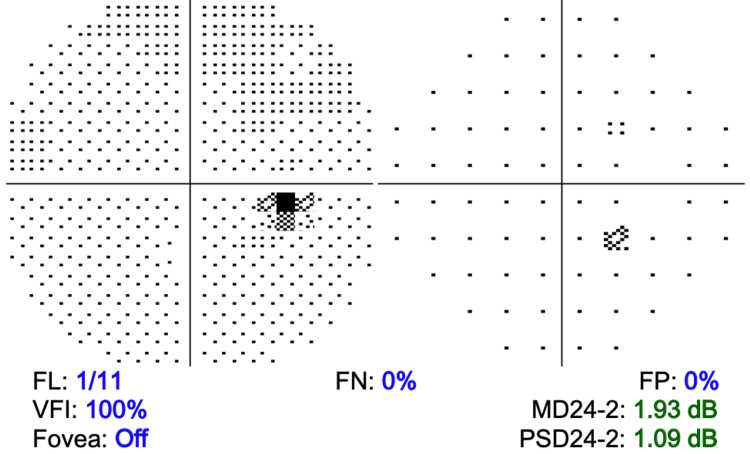
Visual field test of the right eye within normal limits before diagnosis of herpes zoster ophthalmicus. VFI, visual field index; FL, fixation loss; FP, false positive; FN, false negative; PSD24-2, pattern standard deviation 24-2; MD24-2, mean deviation 24-2

**Figure 2:**
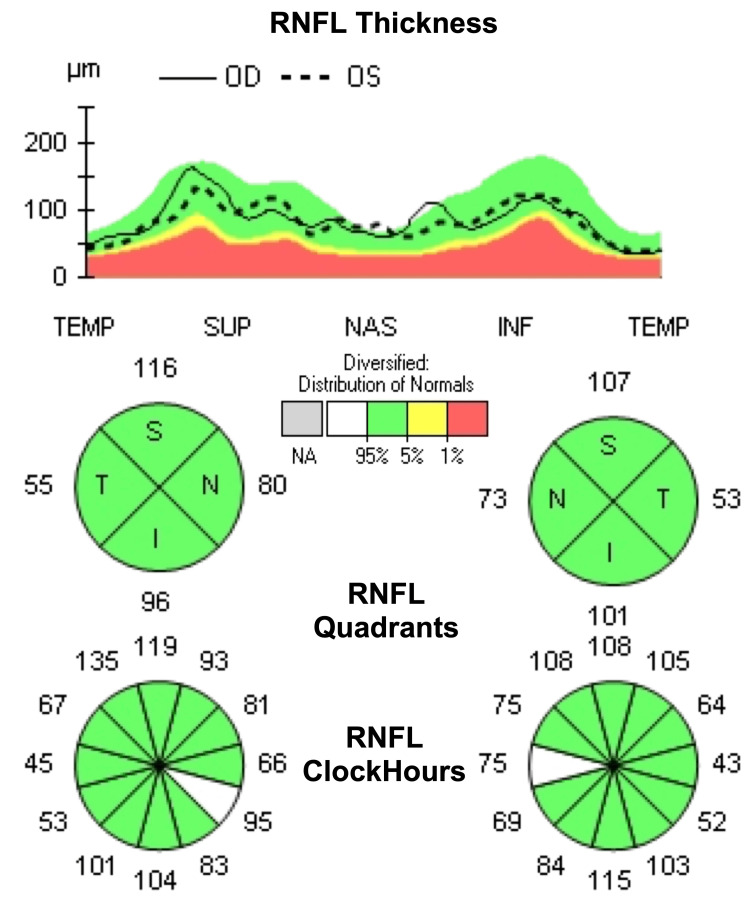
Ocular coherence tomography shows the right eye retinal nerve fiber layer without advanced neuropathy before the diagnosis of HZO. HZO, herpes zoster ophthalmicus; RNFL, retinal nerve fiber layer

Two weeks before arriving at the ophthalmology clinic, the primary care physician initially diagnosed and initiated treatment for HZ with oral valacyclovir (1,000 mg three times daily) and ganciclovir ophthalmic drops (every three hours). The patient had a past ocular history of 1+ nuclear sclerosis and 1+ cortical cataract in both eyes (OU), pinguecula OD, type 2 diabetes mellitus without diabetic retinopathy, choroidal nevus OD, open angle with borderline findings, and low glaucoma risk OU. The patient's visual field testing was within normal limits with no defects (Figure 1), and optical coherence tomography (OCT) showed minimal optic nerve damage before a diagnosis of HZO (Figure 2).

After our initial encounter with the patient, he was referred to the emergency department for imaging studies and instructed to continue the antiviral regimen and start loteprednol etabonate ophthalmic drops (three times daily OD) and erythromycin ointment (two times daily OD). While in the hospital, the patient received magnetic resonance imaging (MRI) of the brain and orbits to rule out other potential causes of OAS. Brain MRI showed enhancement of the cisternal segment of the right trigeminal nerve. Orbital MRI demonstrated enhancement in the right orbital apex in Meckel’s cave and slightly increased enhancement on the right extraocular muscles. Magnetic resonance angiography showed normal arterial evaluation and ruled out possible causes due to thrombosis. Orbital apex syndrome secondary to HZO was then confirmed by viral cultures and polymerase chain reaction testing. While in the hospital, the patient was given oral prednisolone (60 mg daily), prednisolone acetate ophthalmic drops (four times daily OD), and ofloxacin ophthalmic drops (four times daily OD).

At one week follow-up, the right frontal and periorbital region had persistent edema and pain, while the patient complained of diplopia and photophobia. Ophthalmoplegia and restricted extra ocular muscles (EOM) in the right eye remained the same. The patient continued to receive oral prednisolone and maintenance valacyclovir as the mainstay of treatment. Serial ophthalmological examinations were performed to monitor for any signs of worsening visual function. Over the following weeks, the patient’s pain subsided, the vesicular rash resolved, and visual acuity slowly improved. At the seven-week follow-up visit, ophthalmoplegia showed minimal signs of improvement with positive APD, persistent nerve palsy (CN II, III, IV, and VI), and improvement of ptosis of the right eye. On fundoscopic examination, the cup-to-disc ratio was 0.8.

**Figure 3:**
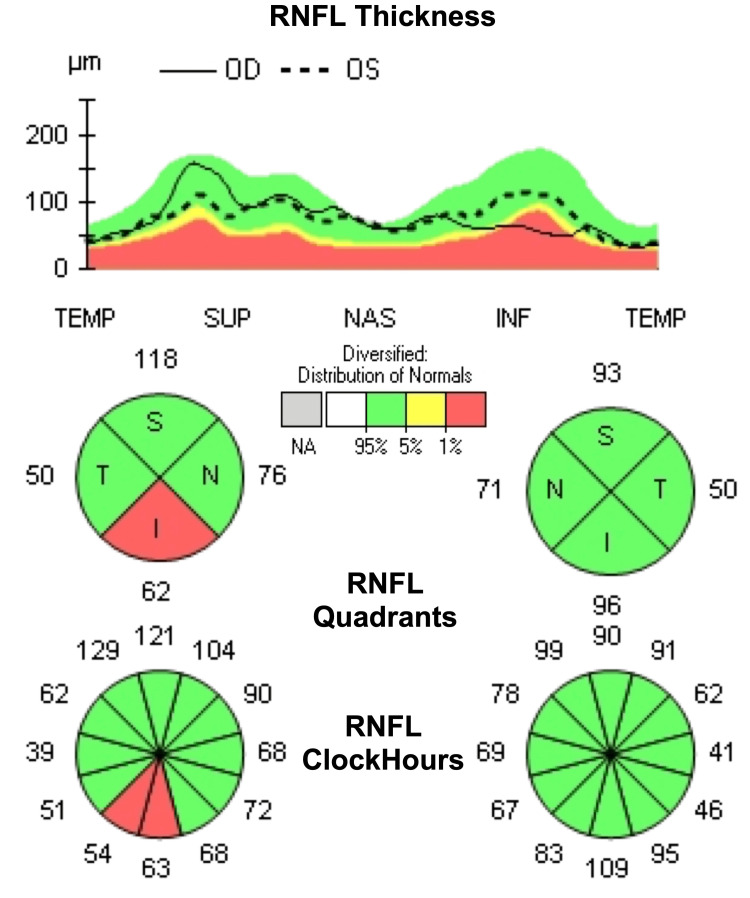
Ocular coherence tomography reveals a decrease in retinal nerve fiber layer thickness in the inferior quadrant of the right eye, one year after the diagnosis of HZO. HZO, herpes zoster ophthalmicus; RNFL, retinal nerve fiber layer

**Figure 4:**
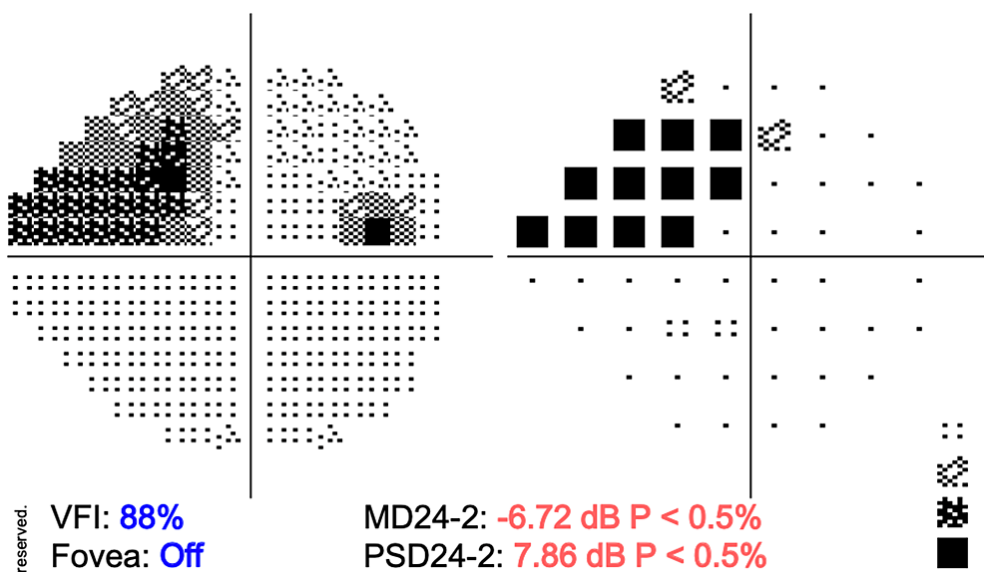
Visual field testing demonstrates a deterioration in the visual field of the right eye one year after the diagnosis of HZO. VFI, visual field index; PSD24-2, pattern standard deviation 24-2; MD24-2, mean deviation 24-2

Evolution was progressively favorable, and after six months, the patient’s right eye regained light reflex and visual acuity improved (best corrected visual acuity [BCVA] 20/60 OD, 20/30-2 OS). Adduction, supraduction, and infraduction had been recovered. After one year, the patient was evaluated for optic nerve damage, and visual field testing showed a progressive increase in the cup-to-disc ratio (0.86 OD, 0.79 OS) (Figure 3) and a decrease in visual field index (VFI) to 88% from the previous 100% (Figure 4).

## Discussion

Oculomotor cranial nerve pareses have been observed in 5% to 31% of patients with HZO [7,8]. OAS is a more extensive and severe complication involving complete ophthalmoplegia with involvement of the V1 branch and optic nerve dysfunction [7]. The causes of OAS include a variety of inflammatory, infectious, neoplastic, vascular, and iatrogenic conditions [7]. Within the infectious etiologies, mucormycosis and aspergillosis are the most common, while varicella zoster virus (VZV) is rarely reported as a cause [7,9,10]. Patients with susceptibility such as diabetes mellitus, alcoholism, malignancy, and immunosuppression should be further evaluated for these conditions [11]. The patient presented in this case had a medical history of diabetes mellitus. In the literature, there are a total of about 20 reported cases describing OAS due to HZO [12,13]. On average, OAS presents about 10 days following zoster rash emergence [8]. This case demonstrated a latency period of 14 days until ophthalmoplegia was confirmed after the initial rash.

Viral reactivation and spread on the surrounding tissues can lead to direct injury or an immune complex-mediated response. Possible pathogenic mechanisms include immune-mediated tissue damage, direct cytopathic effect of the virus, and inflammatory edema causing compression and ischemia [6,8]. The clinical manifestations seen in our patient such as lesions in the face, edema, and conjunctival injection strongly indicated a diagnosis of HZO. The restricted motility, ptosis, and parasympathetic pupillary dysfunction suggested the involvement of the superior and inferior branches of the oculomotor nerve (III) within the superior orbital fissure. MRI confirmed the diagnosis, demonstrating enhancement along the right optic nerve sheath, extraocular muscles, Meckel’s canal, and the cisternal segment of the right trigeminal nerve. The presence of damage affecting both the superior orbital fissure and the optic canal was indicative of OAS. Several studies reported the occurrence of gradual improvement in these findings following treatment [14,15].

Factors that increase the risk of developing HZO-OAS include being over 50 years old, having a weakened immune system, and lacking a history of herpes zoster vaccination [14,16]. Studies evaluating the role of vaccination in the incidence and severity of OAS secondary to HZO are necessary. While there is no universally agreed-upon standard treatment for HZO-OAS, the current therapeutic recommendations are grounded on expert opinion [13,14]. Commonly, a systemic steroid and an oral antiviral have been seen to be effective therapy [16]. Treatment with antiviral agents (acyclovir, valacyclovir, or famciclovir) should be initiated within the first 72 hours after the onset of symptoms to reduce the formation of new vesicles, lessening pain, and preventing viral shedding, thus reducing the risk of recurrence and secondary complications [2,7,8,15]. Lim et al. showed that the delay of initiation of treatment demonstrated poor outcomes, while cases with better prognoses were attributed to early administration of oral antiviral [4]. However, the optimal time for initiation of treatment is within 72 hours from the onset of the rash [4]. The duration of treatment is empirical and has a varying degree across all studies extending from two to six months, depending on recovery [13].

Recuperation from ophthalmoplegia and vision loss is not well known [4]. In a literature review of 20 cases, the resolution rate of ophthalmoplegia was found to be 76.5% of cases within a time frame ranging from two weeks to 1.5 years (mean 4.4 months) [3,7]. This range of time was also observed in our case. Verhaeghe et al. conducted a literature review, revealing that over half of the patients (9/15, 60%) experienced partial improvement in visual acuity, while complete resolution was infrequent (4/15, 26.7%). The prognosis for ocular motility and ptosis, on the other hand, was more positive, with five (33%) patients achieving full recovery and eight (53%) patients showing partial recovery [16].

## Conclusions

Orbital apex syndrome is an infrequent complication of VZV infection and HZO. The occurrence of optic neuropathy and ophthalmoplegia is likely attributed to direct damage to the nerves caused by the virus, along with inflammation and demyelination. Brain and orbital MRI proved to be key in identifying the involvement of orbital apex. Systemic treatment with corticosteroids and antiviral medication managed the ophthalmoplegia; however, limited visual recovery was achieved. Irreversible optic nerve damage is of primary concern one year after infection resolution. Further studies evaluating the outcomes of HZO-OAS, such as optic-neuropathy-induced vision loss, are needed to examine the efficacy of treatment and optimal time frame of administration to reduce comorbidities and sequelae. Timely administration of systemic corticosteroids and systemic antivirals is beneficial for better prognosis. Significant blinding complications of HZO should be of concern in patients with risk factors such as diabetes mellitus, advanced age, and immunodeficiency. Medical practitioners should be diligent in referring patients with HZO to ophthalmologists promptly to improve outcomes.
